# The Capacity of Red Blood Cells to Reduce Nitrite Determines Nitric Oxide Generation under Hypoxic Conditions

**DOI:** 10.1371/journal.pone.0101626

**Published:** 2014-07-09

**Authors:** Marcel H. Fens, Sandra K. Larkin, Bryan Oronsky, Jan Scicinski, Claudia R. Morris, Frans A. Kuypers

**Affiliations:** 1 Children's Hospital Oakland Research Institute, Oakland, California, United States of America; 2 RadioRx, Inc., Mountain View, California, United States of America; 3 Department of Pediatrics, Emory University School of Medicine, Atlanta, Georgia, United States of America; Albany Medical College, United States of America

## Abstract

Nitric oxide (NO) is a key regulator of vascular tone. Endothelial nitric oxide synthase (eNOS) is responsible for NO generation under normoxic conditions. Under hypoxia however, eNOS is inactive and red blood cells (RBC) provide an alternative NO generation pathway from nitrite to regulate hypoxic vasodilation. While nitrite reductase activity of hemoglobin is well acknowledged, little is known about generation of NO by intact RBC with physiological hemoglobin concentrations. We aimed to develop and apply a new approach to provide insights in the ability of RBC to convert nitrite into NO under hypoxic conditions. We established a novel experimental setup to evaluate nitrite uptake and the release of NO from RBC into the gas-phase under different conditions. NO measurements were similar to well-established clinical measurements of exhaled NO. Nitrite uptake was rapid, and after an initial lag phase NO release from RBC was constant in time under hypoxic conditions. The presence of oxygen greatly reduced NO release, whereas inhibition of eNOS and xanthine oxidoreductase (XOR) did not affect NO release. A decreased pH increased NO release under hypoxic conditions. Hypothermia lowered NO release, while hyperthermia increased NO release. Whereas fetal hemoglobin did not alter NO release compared to adult hemoglobin, sickle RBC showed an increased ability to release NO. Under all conditions nitrite uptake by RBC was similar. This study shows that nitrite uptake into RBC is rapid and release of NO into the gas-phase continues for prolonged periods of time under hypoxic conditions. Changes in the RBC environment such as pH, temperature or hemoglobin type, affect NO release.

## Introduction

The principal function of red blood cells (RBC) is delivery of oxygen to all tissues in the body. Key to adequate oxygen delivery is blood flow, which is determined by the dilation and contraction of blood vessels. Vascular tone and the maintenance of vascular homeostasis are regulated in part by nitric oxide (NO). The physiological importance of the interactions of nitrite and NO with hemoglobin was established with the identification of NO as the endothelium-derived relaxation factor (EDRF) [1], [2]. Since RBC play a crucial role in oxygen delivery, it is logical to assume that they have an intrinsic capacity to be an essential player in the endocrine regulation of vascular tone. However, the exact mechanism by which intact RBC under physiologic conditions and different oxygen tensions play their role in NO signaling remains mainly an unanswered question. In the presence of oxygen, NO is generated by the enzymatic conversion of L-arginine by nitric oxide synthases (NOSs), which catalyze the synthesis of NO and formation of citrulline from the reaction with L-arginine and nicotinamide adenine dinucleotide phosphate (NADPH) [3]. Three different NOSs have been identified: nNOS, iNOS and eNOS (NOS 1–3). Whereas nNOS predominantly functions as a neurotransmitter and iNOS contributes to host defense mechanisms against pathogenic microorganisms, eNOS plays a key role in the regulation of vascular tone [3], [4]. The enzyme eNOS is also identified in the membranes of RBC and could play a role in the formation of NO [5], [6]. During hypoxia, however, the activity of eNOS is repressed and NO formation is managed by a different mechanism [7], [8]. It has been reported that xanthine oxidoreductase (XOR) present in the RBC, generates NO from nitrite [9], [10], mainly at lower pH and under hypoxic conditions. In addition, aldehyde oxidase (AO) has been described to contribute to RBC-mediated NO formation [11]. However, it seems beyond dispute that the nitrite reductase capacity of hemoglobin is of major importance in the regulation of vascular tone under hypoxic conditions. Deoxyhemoglobin has the ability to convert nitrite (NO_2_^-^) to NO, providing a major source for NO in the circulation. Under strictly anaerobic and physiological conditions, addition of an excess of nitrite result in (equal molar) formation of methemoglobin (HbFe^3+^) and iron-nitrosyl hemoglobin (HbNO) [12], [13]. In the first reaction, deoxyhemoglobin reacts with nitrite to form NO and methemoglobin, followed by the reaction of NO with deoxyhemoglobin to result in HbNO [13]. The presence of oxygen and oxyhemoglobin leads to augmented HbFe^3+^ formation [14]. The allosteric tetrameric conformation for deoxyhemoglobin is a ‘tensed’ or ‘T-state’. This conformation is transformed as a result of the nitrite to NO conversion, to a ‘relaxed’ or ‘R-state’ conformation because of ligation of NO to Hemoglobin. The increase in R-state hemoglobin is associated with a higher rate constant and greater nitrite reductase activity [13]. Biological sources of nitrite for this reaction include the conversion of dietary nitrate by commensal symbiotic oral flora, and oxidation of intravascular NO produced by NOSs [15], [16]. Dysregulation of NO metabolism is a hallmark of many diseases and events such as cardiovascular arrest and sickle cell disease. Intravascular hemolysis, whether high or low levels, will release arginase, free hemoglobin and other contents from the red cell, which breaks down arginine, thereby lowering the available substrate for eNOS [17]. In addition, cell free hemoglobin reacts with NO. Research has been mainly focused on the dysregulation of arginine-to-NO metabolism caused by RBC. However, modifications of hemoglobin could play a major role, particularly under low oxygen conditions. Importantly, the role of the intact RBC to generate NO from nitrite under physiologic conditions has received limited attention, as it involves a number of pathways including uptake of substrate (nitrite), conversion of substrate to product (NO), reaction of product with many entities in the RBC, and finally the release of NO into the environment. Evaluating these pathways is further complicated by the fact that nitrite is rapidly oxidized to nitrate under normoxic conditions, affecting the substrate levels for the RBC induced reductase activity. In this study, we investigated the nitrite reductase kinetics and capacity of RBC with physiological hemoglobin concentrations in various conditions and with different hemoglobin types. By evaluating the intact RBC at physiologic concentrations as the “enzymatic activity” to generate NO from nitrite, has advantages. The measurement of the conversion from substrate to product takes into account all steps in the process: uptake of substrate, conversion to NO inside the RBC, release of NO from the cell, and the changes in the RBC that result from this process. We used a novel, reproducible method to study RBC-mediated nitrite-to-NO reduction, which mimics the NO detection system that is used in clinical practice to detect NO in exhaled air. This method allowed detection of NO release under various conditions at physiological hemoglobin concentrations.

## Materials and Methods

### Sample preparation

For RBC and hemolysate samples, normal (HbAA) and sickle blood (HbSS) was collected in EDTA/K3 tubes after written consent with institutional (CHRCO) IRB approval. Umbilical cord blood, from anonymously donated placentas, was collected in heparin tubes with institutional IRB approval and processed within a day of collection. Percentage HbF was determined using High Performance Liquid Chromatography (HPLC). Blood samples where characterized by a complete blood count (Advia 120 hematology analyzer, Siemens, Munich, Germany) and RBC were collected by centrifugation, removal of plasma and buffy coat by twice washing in HEPES buffered saline (HBS). Packed RBC were diluted with HBS containing 2% bovine serum albumin (HBS-BSA, Sigma-Aldrich, St. Louis, MO, USA), to a 11–12 g/dL hemoglobin (equivalent to 4.0±0.1×10^6^ RBC/µl) suspension. Unless indicated otherwise HBS at pH 7.4 was used. For pH studies, HBS with a pH of 6.8, or 5.5 was used representing clinically severe acidosis in the blood or tissue ischemia [10], respectively. RBC samples were either run as intact RBC (within 1 day after withdrawal) or as hemolysates. Hemolysates were RBC samples that were fully lysed by applying 3 freeze-thaw cycles. Hemolysis of RBC was measured by the release of hemoglobin in the medium from RBC at regular time points during the prolonged incubation in the tonometer. Preliminary experiments indicated that addition of 2% albumin to the incubation buffer greatly reduced hemolysis to less than 1% when RBC at 30% hematocrit (hct) were incubated under regular stirring for 100 minutes. All experiments reported were performed in albumin containing HBS.

### NO release & nitrite influx

#### NO donors

Two NO donors with different release kinetics were diluted in HBS-BSA 2% or added to the blood samples. Rapidly dissociating Diethylamine NONOate (DEA NONOate; Cayman Chemical, Ann Harbor, MI, USA) was diluted to final concentrations of 20, 50 µM and 100 µM. Slower dissociating Spermine NONOate (Sper NONOate; Cayman Chemical) was diluted to final concentrations of 50 and 250 µM. *Blood/hemoglobin samples*. In a tonometer (Instrumentation Laboratory, Bedford, MA, USA), blood samples (0.5 ml) were incubated in a glass cuvette under a constant gas flow (180 ml/minute), of nitrogen or normal air resulting in a partial oxygen tension of 0 or 160 mmHg, respectively. For the carbon monoxide (CO) experiments, a mixture of CO (20 ppm) balanced with N_2_ was used. The tonometer was set for a continuous set of spinning cycles to ensure proper gas exchange at the buffer gas interphase and was used at a constant temperature (37°C unless otherwise indicated). RBC were exposed to CO, 20 minutes before the addition of nitrite and start of the general protocol.

Incubation of blood samples with NO, 30 minutes prior to addition of nitrite and the general protocol, was obtained by the addition of 25 µl of 100 µM DEA NONOate to a final concentration of 5 µM. NO donor-mediated NO release from the tonometer was checked before proceeding to the general protocol to prevent false-positive NO readings.

#### Nitrite incubation

**Figure 1:**
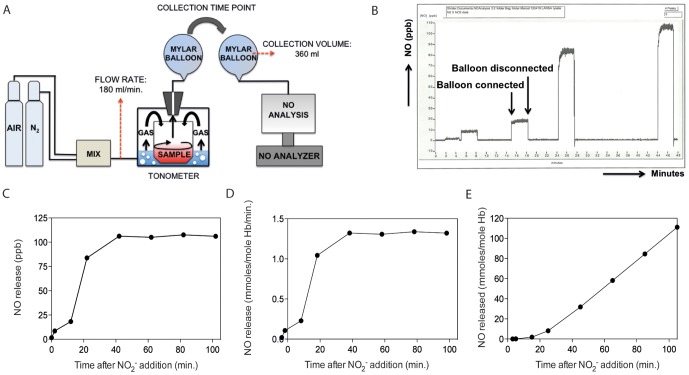
A novel method to determine nitrite reductase capacity of patient blood samples. (A) Cartoon depicting the set-up for the newly designed *in vitro* nitrite reductase detetion method of blood samples. Single gas or a mixture of gases with defined composition is led through a tonometer at a set flow rate of 180 ml/minute. Inside the tonometer, a glass cuvette, containing 500 µl blood sample, is heated to a set temperature and stirred constantly to promote rapid equilibration at the water-gas interphase. Gas leaves the tonometer solely through an opening in the lid and is collected in mylar balloons at predetermined time points for 2 consecutive minutes. NO concentration present in the mylar balloons is measured in a Nitric Oxide Analyzer by detecting chemiluminescence resulting from the reaction of ozone with NO. (B) Typical example of NO detected in balloons, collected in time, using filtered room air as background reference. Concentrations of NO released into balloons are recorded as parts per billion (ppb) (C). Next, the NO concentration is converted to moles per minute and corrected for the hemoglobin concentration present in the tonometer, in order to obtain the NO release rate (D). Finally, the total amount of NO released from the tonometer, during an entire run, could be calculated per mole hemoglobin (E).

Nitrite incubations were initiated after 5 minutes initial incubation of the RBC mixture in the tonometer, to equilibrate the samples to temperature and gas conditions. Before addition of nitrite, gas was collected for background NO values. Next, 100 µl of a sodium nitrite solution (Sigma-Aldrich) was added to the blood sample by pipetting directly into the cuvette using an elongated pipet tip. This allowed addition without changing the gas-state in the tonometer. At set time points (generally after 0, 10, 20, 40, 60, 80 and 100 minutes) gas outflow samples were taken to measure NO release using mylar balloons, with gas collection for 2 minutes. The NO in the mylar balloons was determined similar the to analysis of NO in exhaled air by the Sievers nitric oxide analyzer (NOA 280, GE Water & Process Technologies, Trevose, PA, USA) (See Figure 1B). All NO peaks were recorded by the NOAnalysis™ software. This allowed peak quantification (see example Figure 1C). Finally, NO values collected are used to calculate the NO release rate (see example Figure 1D) and to calculate the sum of total mole NO (see example Figure 1E) released per mole hemoglobin during the run.

#### Nitrite influx

For the nitrite influx studies, 8 µl was collected from the mixture immediately after adding nitrite, followed by sampling the same volume at 5, 10, 20, 40 and 100 minutes during a run. Sample collection was performed without changing the gas-state in the tonometer. The samples were spun down immediately upon collection and the supernatant and RBC pellet, after being washed with HBS, were stored at −20°C until nitrite was measured. The nitrite concentration was measured, at 550 nm on a Spectramax 340, by the colorimetric Thomson-Griess assay (Sigma-Aldrich) [18]. Curve fit and regression was performed using KaleidaGraph (Synergy Software, Reading, PA, USA).

### Statistical analysis

Data were statistically analyzed by nonparametric 2-way ANOVA with Bonferroni post-test using GraphPad Prism 6 for Mac OS X (GraphPad Software, San Diego, CA, USA).

## Results

### NO donors show first order NO release

We have developed a modified system to study NO release *in vitro* from blood samples. NO was determined by its release into the gas-phase similar to measurement of NO in exhaled air, allowing study of the reductase capacity of RBC under various conditions. To validate the measurement of NO from the tonometer, we analyzed NO released from two different NO donors that generate NO with first order kinetics; diethylamine (DEA) NONOate, a rapid dissociating molecule and Spermine (Sper) NONOate, a much slower dissociating NO donor (release half-life at physiological conditions 2.1 and 39 min, respectively [19]). Two concentrations of DEA NONOate under constant nitrogen flow were measured, and an initial high spike of NO was detected, decreasing rapidly at subsequent time points for both concentrations of DEA NONOate (Figure S1A in File S1). As expected, when Sper NONOate was added under nitrogen flow, a much slower release of NO was observed as compared to DEA NONOate, (Figure S1B in File S1). This showed that indeed NO released in the gas-phase could be detected as expected. When the highest concentration of either NONOate was added to RBC hemolysates, less than 2% of NO release was detected as compared to release in buffer alone (Figure S1C in File S1). These data indicate that our method efficiently detects the release of NO in the gas-phase and also indicates that the presence of RBC derived material (hemolysate) dramatically reduces the NO that is measured as released in the gas-phase, likely as the result of the reaction of NO with components in cytosol or membrane of RBC.

### RBC-mediated nitric oxide release detection at 5 mM nitrite at physiological hemoglobin concentration

**Figure 2:**
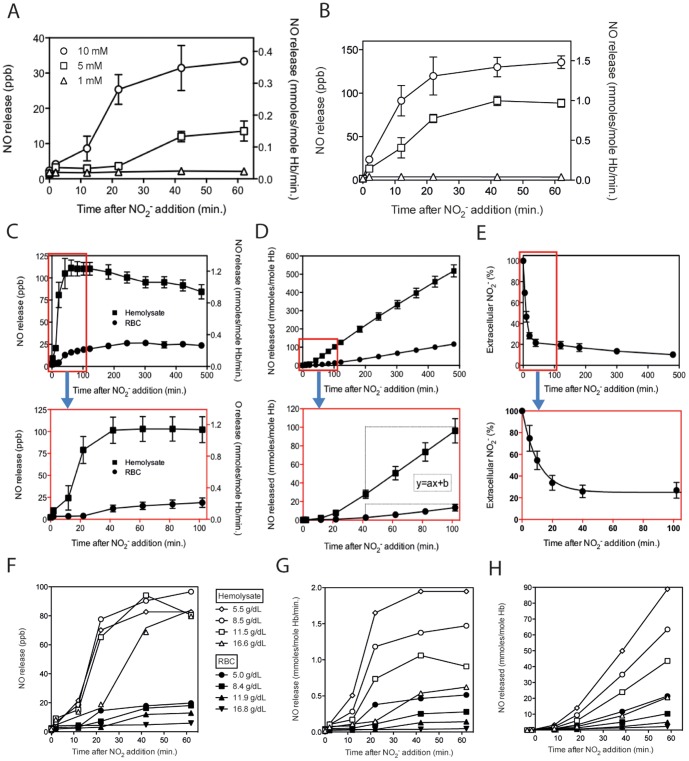
Nitrite-induced NO release and cellular nitrite influx under hypoxia. Blood samples containing approximately 12/dL hemoglobin were incubated with 1, 5 and 10 mM nitrite in order to determine the detection limit. Both normal RBC and hemolysate samples showed detectable NO release at 5 and 10 mM and undetectable NO release at 1 mM nitrite (A and B). However, for RBC samples, NO release at 5 mM showed parts-per-billion (ppb) signals unambiguous above detection background levels (A), making this concentration suitable for induction of NO release in our method. To determine the most appropriate time frame in which to collect NO release data, RBC and hemolysate samples were run with 5 mM nitrite for 8 hour at 37°C, under strict hypoxia. After an initial delay in NO release, a relatively constant NO release level was detected after 40 minutes for both RBC and hemolysate samples, which was reported as NO signal (ppb) measured and release rate in (C). Cumulative NO release in time showed linear kinetics from about 40 minutes on, allowing calculation of the constant slope (a measure for NO release rate), which is 0.179 (R^2^ = 0.997) for RBC, and 1.141 (R^2^ = 1.000) for hemolysates (D). Based on these results we decided to run all samples for 100 minutes. Nitrite influx into RBC was considerably rapid and within 40 minutes after addition only about 20% of the total nitrite added was detected in the extracellular compartment, indicating that 80% was transported inside (E). Since total RBC volume was only 35%, nitrite was transported into the cells, where it was likely consumed by hemoglobin. A curve fit following y = a+b*exp-kt (R^2^ = 0.996) resulted in a constant of 0.095 and reached a maximum uptake of approximately 75% of extracellular nitrite. Incubation of RBC and hemolysate samples with 5 mM at various hemoglobin concentrations showed that increasing hemoglobin concentrations resulted in comparable released NO values (ppb) (F). Correction for hemoglobin concentration showed that, both for RBC and hemolysates, samples with lowest hemoglobin concentration were highest in NO release rate (G) and consequently also in total NO released per mole Hb (H). A & B: RBC n = 3, Hb 11.7±0.3 g/dL, hemolysates: n = 3, Hb 11.9±0.2 g/dL, C: RBC n = 3, Hb 11.7±0.1 g/dL, hemolysates: n = 4, Hb 11.6 g/dL±0.3 g/dL, D: RBC n = 32 (14 different donors), Hb 11.6±0.3 g/dL, hemolysates: n = 29 (14 different donors) Hb 11.6±0.3 g/dL, E: influx curve fit; y = 25.0+76.2*exp(-0.095*t), R^2^ = 0.996

To determine the minimal nitrite concentration that would lead to detectable NO release levels, a range of nitrite concentrations was added to RBC and hemolysate samples. For RBC and hemolysate samples 5 mM nitrite appeared to present the approximate lower limit leading to detectable NO levels in normal RBC samples during the time frame of the experiment (Figure 2A and 2B). Hemolysates showed a higher NO release rate, even though hemoglobin concentration in the RBC and hemolysate samples was matching. Incubation of hemolysates with 1 mM resulted in undetectable NO release levels (Figure 2B). Based upon these results, 5 mM nitrite was used in all experiments described unless otherwise mentioned. Transport of extracellular nitrite into RBC was virtually 100% for 1 mM, 75% for 5 mM and around 50% for 10 mM (Figure S2A in File S1). The NO release from RBC as compared to NO release from hemolysates with the same hemoglobin concentration, was significantly different. Total NO release measured in RBC was much lower compared to hemolysate samples (containing the exact same components as RBC samples) that were incubated with the same concentration of nitrite. Hemoglobin density likely affects NO scavenging by proximate hemoglobin molecules, possibly causing decreased NO release from the cuvette. To confirm that hemolysate is superior to RBC with respect to NO release, we mixed intact RBC with hemolysate. When 5 or 10% of RBC in the RBC samples were replaced with hemolysate, equivalent based on hemoglobin content, the RBC samples mixed with hemolysates showed increased NO release in a hemolysate concentration dependent matter (Figure S3 in File S1).

To determine the most favorable time frame in which blood samples should be exposed to nitrite, RBC and hemolysate samples were incubated with 5 mM nitrite for 8 hours under strict hypoxia. After an initial delay, the nitrite reductase activity seemed to follow apparent first order kinetics since NO release is constant and linear for both RBC and hemolysate samples. The NO release rate and the total cumulative NO released are depicted in Figure 2C and 2D, respectively. Additionally, cumulative NO release data allowed linear regression, the constant slope (also a measure for rate) for RBC was 0.179 (R^2^ = 0.997), and 1.141 (R^2^ = 1.000) for hemolysates.

In the case of intact RBC, the availability of nitrite to the reducing interior of the RBC is essential for NO generation to take place. The influx kinetics of nitrite, when added to the RBC sample in the tonometer, showed a rather swift transport into the RBC (Figure 2E). Applying a curve fit for equilibrium diffusion in a 2 pool model ([nitrite]t/([nitrite]t_0_ = A+B*exp-kt) rendered a excellent curve fit (R^2^ = >0.95), and pool sizes that reflected the equilibrium level, but did not match the pool sizes as predicted by the volumes of RBC and medium. At 35% hematocrit the extracellular nitrite concentration dropped to approximately 25%, of the originally concentration of nitrite, added after 100 minutes. A simple diffusion equilibrium predicts that 65% would maintain in the medium. The nitrite influx for samples with a hemoglobin concentration of 5.0, 8.4, 11.9 and 16.8 g/dL (equivalent to 15, 25, 35 and 50% hematocrit, respectively), confirmed this finding, as approximately 65, 40, 25 and 5% of the total nitrite added to these samples, remained in the extracellular compartment after 60 minutes (Figure S2B in File S1). These data show a preferential uptake of nitrite into RBC. After 8 hours incubation at a 35% hematocrit, the extracellular nitrite level dropped even further to around 10%, indicating that after the initial rapid influx into RBC, nitrite was further ‘consumed’ by the RBC during the following 7.5 hours and compensated by additional influx. When 5 mM nitrate instead of nitrite was added to RBC under hypoxic conditions, no NO release was detected and no nitrate influx into the RBC was found (data not shown).

Incubation of 5 mM nitrite with RBC and hemolysate samples with a range of hemoglobin concentrations surprisingly showed larger NO release with decreasing hemoglobin concentrations for RBC samples and comparable NO for all hemolysate concentrations except 16.6 g/dL, which was noticeably delayed (Figure 2F). These differences became more apparent when results were corrected for hemoglobin concentration and converted to NO release rate (Figure 2G) and total NO released during 100 minutes (Figure 2H).

Together these data show that our setup efficiently monitors the release of NO from the reduction of nitrite into the gas-phase by either intact RBC or RBC components (hemolysate) at RBC concentrations similar to those found in the circulation. Nitrite is rapidly and preferentially taken up by RBC, which act like a sink for nitrite. The release of NO into the gas-phase under our conditions is due to the reduction of nitrite by the RBC, and the release of NO into the gas-phase by RBC seems counteracted by the presence of RBC or hemolysate, suggesting a reaction of the formed NO with the RBC components. These basic measurements formed the starting point to alter the incubation conditions to further evaluate the nitrite reductase activity of RBC.

### Oxyhemoglobin lacks NO release and introduction of oxygen during a run dramatically reduces NO release

**Figure 3:**
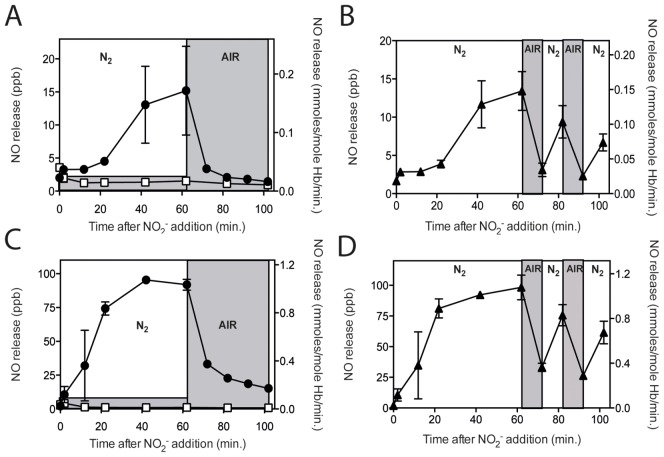
Oxyhemoglobin lacks NO release and introduction of oxygen during a run dramatically reduces NO release. When RBC and hemolysates in the tonometer were exposed to normal air (20.8% O_2_) instead of N_2_, no NO release upon 5 mM nitrite addition could be detected during the entire run (A & C). Converting deoxyhemoglobin to oxyhemoglobin after 62 minutes, by replacing the N_2_ stream by air, resulted in a dramatic drop in detectable NO released from the tonometer for both RBC and hemolysates (A & C). Switching back to N_2_ after 10 minutes exposure to air recovered the NO release to about 75–80% (B & D). Repeating this N_2_-air-N_2_-cycle again showed a severe drop in NO release followed by a significant recovery when N_2_ was reapplied. □ exposed to air from t = 0 minutes, • exposed to air from t = 62 minutes, ▴ exposed to air from t = 62–72 and 82–92 minutes. A: RBC air n = 2, Hb 11.9±0.2 g/dL, RBC N_2_-air n = 3, Hb 11.6±0.5, B: RBC N_2_-air-N_2_ n = 3 Hb 11.7±0.3, C: hemolysate air n = 2, Hb 11.7±0.2, hemolysate N_2_-air n = 2, Hb 11.7±0.2, D: hemolysate N_2_-air-N_2_ n = 3, Hb 11.8±0.1

When blood samples were exposed to normal air with 20.8% oxygen (160 mmHg partial oxygen pressure) before the addition of 5 mM nitrite, to allow only oxyhemoglobin species to be present, no NO release could be detected for either RBC or hemolysate samples (Figure 3A and 3C). Reducing the oxygen percentage to 3.5% (30 mmHg, the partial oxygen pressure oxygen at which 50% of hemoglobin is saturated with oxygen for normal RBC), rendered similar results as compared to air; no NO release (data not shown). It has to be taken into account that nitrite will be oxidized to nitrate (NO_3_^-^) during these experiments under air. Measuring both nitrite and nitrate under our conditions showed that after 40 minutes under normal air approximately 40% of nitrite was converted to nitrate (Figure S4 in File S1). While the decrease in substrate for the reductase activity will affect the generation of NO, this reduction is very unlikely to lead to the total inhibition observed of NO for3mation. Based on the released NO measured in the balloons, of the substrate (nitrite) available in the RBC, only 1–2% is converted to NO after 60 minutes. However, a substantial fraction of formed NO reacts and remains inside the tonometer. When 2.5 mM nitrate was added to the mixture with nitrite under nitrogen, no reduction in NO formation was observed. Nitrate is not taken up by the RBC indicating that the presence of Nitrate in the external medium formed under incubation under air did not affect the reductase activity. Changing the partial oxygen tension during an experiment showed that this decrease in reductase activity could be at least partly restored by switching from 160 mmHg partial oxygen pressure back to no oxygen. Nitrite was incubated with RBC for 60 minutes under hypoxic conditions (nitrogen) followed by switching to air. The released NO level dropped dramatically as soon as oxygen was present. Nitric oxide levels decreased about 80% compared to the NO release measured at 60 minutes for RBC samples (Figure 3A) and, about 65% for the NO release measured at 60 minutes for hemolysates (Figure 3C), and kept decreasing when the airflow continued. When air was replaced after 10 minutes with nitrogen, NO release levels were largely restored, albeit that the 60 minutes NO release level was not reached (Figure 3B and 3D). After every nitrogen-to-air-to-nitrogen cycle NO release was partly restored, but the original level was not reached. Addition of nitrite restored NO formation (Figure 3D). These data show that the presence of oxygen negatively affected the reductase action of the RBC by reducing the substrate for the reaction, but did not affect the ability to reduce nitrite to NO.

To investigate a possible immediate NO release burst upon the switch between exposure to air and nitrogen we also performed 2 minutes air-N_2_ (oxy-deoxy)-cycles with constant collection of outflow gas. Interestingly, this shows that shorter exposure to nitrogen rather than air decreased the NO release signal (Figure S5 in File S1). The cycles between air and nitrogen will lead to the formation of HbFe^3+^. Comparison of nitrite incubation with commercially purchased purified hemoglobin AA, consisting of mere HbFe^3+^, showed a NO release of about 50% of the NO release detected for hemolysates under the same conditions at the same concentrations (Figure S6 in File S1).

**Figure 4:**
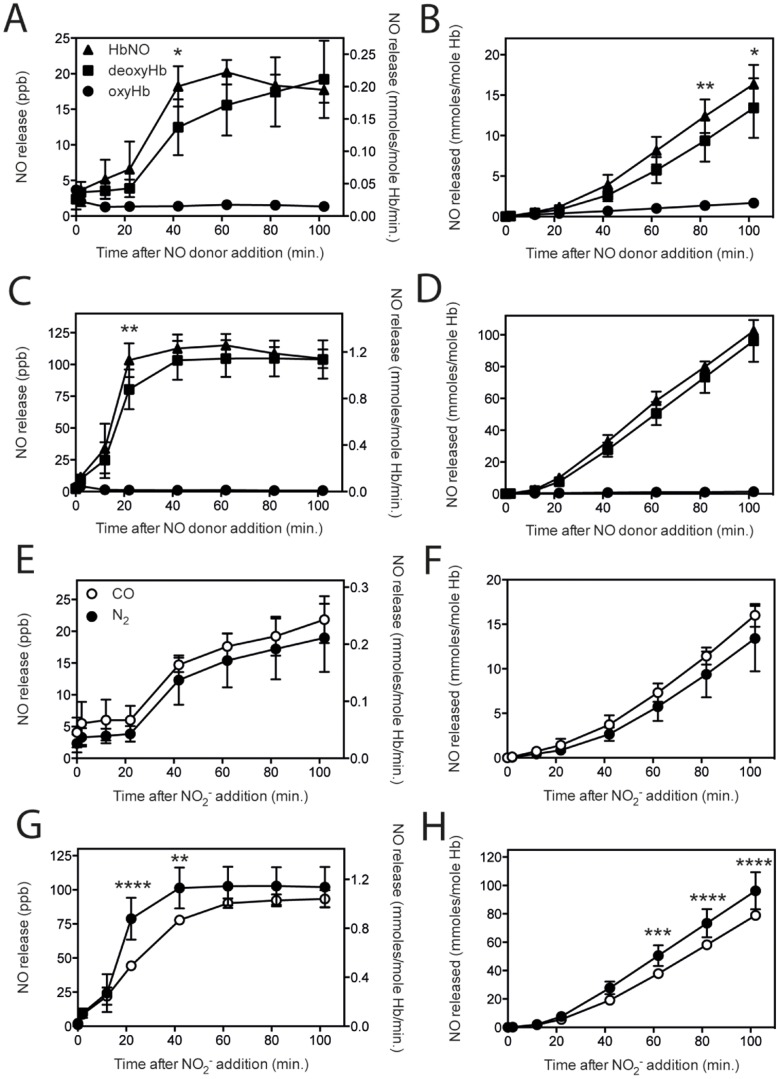
NO or CO-mediated R-state hemoglobin does not induce increased NO release. To study the nitrite reductase capacity of hemoglobin when in a R-state conformation, RBC and hemolysate samples were exposed to with NO and CO before addition of 5 mM nitrite. Iron-nitrosyl-hemoglobin, prepared by addition of an excess of NO donor DEA NONOate (100 µM) under hypoxic conditions, showed matching NO release results compared to deoxyhemoglobin for both RBC and hemolysates reducing rate and total NO released (A–D). As shown in Figure 3, nitrite addition to oxyhemoglobin did not result in NO release. A second way to push hemoglobin into a R-state conformation under hypoxic conditions is by saturation with CO, creating carboxyhemoglobin. Addition of 5 mM nitrite to RBC containing carboxyhemoglobin, showed comparable NO release results for CO exposed and N_2_ exposed samples (E and F). Conversely, hemolysates exposed to CO showed a significant lower NO release when compared to N_2_ (G and H). A & B: RBC oxy n = 2, Hb 11.9±0.2 g/dL, deoxy (N_2_) n = 32, Hb 11.6±0.4 g/dL, HbNO n = 3, Hb 11.7±0.3 g/dL, C & D: hemolysates oxy n = 2 Hb 11.7±0.2 g/dL, deoxy (N_2_) n = 29, Hb 11.6±0.3 g/dL, HbNO n = 4 Hb 11.8 g/dL±0.1, E & F: RBC CO n = 3, Hb 11.7±0.5 g/dL, N_2_ n = 32, Hb 11.6±0.3 g/dL, G & H: Lysates: CO n = 4, Hb 11.8±0.1 g/dL, N_2_ n = 29, Hb 11.6±0.3 g/dL, Statistics: *P<0.05, **P<0.01, ***P<0.001, and ****P<0.0001.

The slower initial release of NO after addition of nitrite suggested that the release of NO was counteracted by the reaction of NO with cellular components. To explore this, we added a limiting amount of NO relative to hemoglobin, but enough to at least partly saturate these expected NO binding sites, including the generation of HbNO [20]. We used a final concentration of 5 µM DEA NONOate in 500 µl containing 2000 µM Hb or a ratio of approximately 1 NO per 400 hemoglobin entities or 1 per 1600 heme entities. NO treatment did not affect the uptake of nitrite into RBC (Figure S7 in File S1). Comparison of NO release under nitrogen with NO incubated RBC or hemolysate before addition of 5 mM nitrite, showed that pre-incubation with NO significantly accelerated the release in the initial phase (Figure 4A and 4B). After 100 minutes either NO treated or untreated RBC rendered similar NO release. Nitric oxide pre-incubated hemolysates showed a similar, be it smaller increase in NO release (Figure 4C and 4D). The rate in the linear phase (between 40 and 100 minutes time points) of NO release from RBC samples was 0.179 (R^2^ = 0.997) for not NO treated RBC and 0.208 (R^2^ = 1.000) for NO treated RBC. For hemolysates these rates were 1.141 (R^2^ = 1.000) and 1.150 (R^2^ = 0.999), respectively.

### The effect of carbon monoxide-mediated, R-state hemoglobin on NO release

To examine whether R-state conformation of hemoglobin, induced by carbon monoxide (CO) rather than O_2_, would affect nitrite reduction under hypoxic conditions, blood samples were exposed to CO prior to the addition of nitrite. CO treatment did not affect the uptake of nitrite into RBC (Figure S7 in File S1). When blood samples were saturated by incubation with CO (balanced with N_2_) prior to nitrite addition, carboxyhemoglobin is formed. Incubation of CO treated RBC did not result in a significant difference as compared to non-treated RBC (Figure 4E and 4F). Similar incubation with hemolysate showed a lower release of NO in CO treated samples (Figure 4G and 4H). The rate in the linear phase of NO release from for CO treated RBC was 0.205 (R^2^ = 0.997) and for hemolysates this was 0.998 (R^2^ = 1.000).

Together these data show that the presence of oxygen has a dramatic effect on the release of NO by RBC. A switch back to deoxygenating conditions partly restored reductase activity, augmented by the addition of nitrite to compensate the loss of nitrite to nitrate. Neither CO or NO treatment affected the uptake of nitrite into the RBC. Modification of hemoglobin by CO moderately affected the formation, and release of NO from RBC. Modification of cellular components with NO affected the nitrite-induced release of NO from RBC, in particular in the first phase of the incubation.

### Decreased pH leads to increased nitrite-to-NO reduction

**Figure 5:**
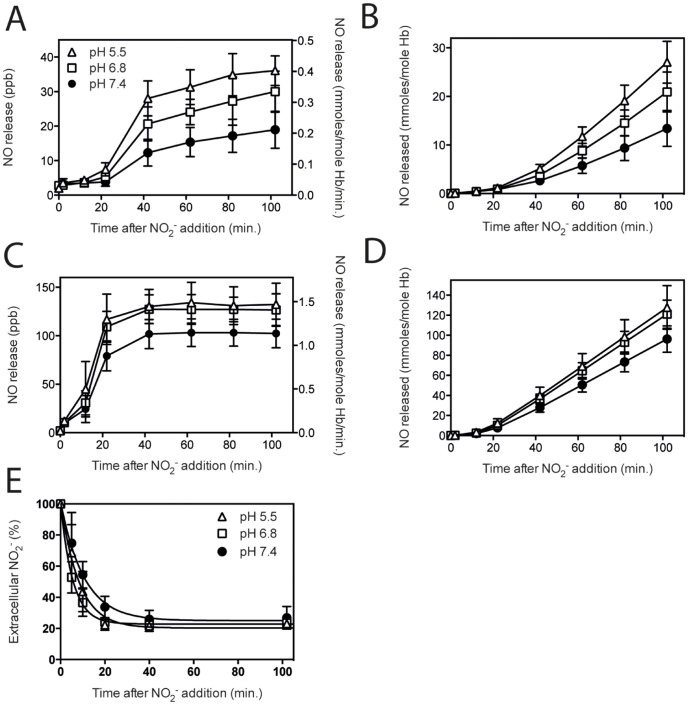
Decreased pH leads to increased nitrite-to-NO reduction. Clinical severe acidosis and tissue ischema was mimicked by incubation of RBC and hemolysates in an environment with reduced pH. Under hypoxia and after addition of 5-mediated NO-release showed a pH-dependent increase. A lower pH resulted in an increased NO release rate (A & C) and therefore total release in time (B & D). Nitrite influx into RBC was also affected by lowering the pH. Both pH 6.8 and pH 5.5 demonstrated a slightly faster nitrite transport into RBC (E). A & B: RBC pH 5.5 n = 3, Hb 11.7±0.3, pH 6.8 n = 3, Hb 11.6±0.1 g/dL, pH 7.4 n = 32, Hb 11.6±0.4, C & D: Hemolysates pH 5.5 n = 4, Hb 11.7±0.2, pH 6.8 n = 4, Hb 11.8±0.2, pH 7.4 n = 29, Hb 11.6±0.3, E: influx curve fits: pH 5.5 y = 20.3+81.3*exp(−0.121*t); R^2^ = 0.990, pH 6.8 y = 22.8+77.0*exp(−0.182*t); R^2^ = 0.999, pH 7.4 y = 25.0+76.2*exp(−0.095*t); R^2^ = 0.996 Statistics: *RBC NO release rate*: pH 7.4 vs. pH 6.8; *** after 42 and 62 minutes, **** after 82 and 102 minutes, pH 7.4 vs. pH 5.5; **** after 62–102 minutes, 6.8 vs. 5.5; * after 42–82 minutes. *RBC total NO release*: pH 7.4 vs. pH 6.8; * after 62 minutes, **** after 82 and 102 minutes, pH 7.4 vs. pH 5.5; **** after 62–102 minutes, 6.8 vs. 5.5; ** after 82 minutes, **** after 102 minutes. *Hemolysate NO release rate*: pH 7.4 vs. pH 6.8; *** after 22 minutes and ** after 42–102 minutes, pH 7.4 vs. pH 5.5; * after 12 minutes, **** after 22 and 62 minutes, *** after 42, 82 and 102 minutes. *Hemolysate total NO release*: pH 7.4 vs. pH 6.8; *** after 62 minutes, **** after 82 and 102 minutes, pH 7.4 vs. pH 5.5; ** after 42 minutes, **** after 62, 82 and 102 minutes. *Influx*: pH 5.5 vs. pH 6.8; * after 5 minutes, pH 6.8 vs. pH 7.4, **** after 5 minutes, and *** after 10 minutes. *P<0.05, **P<0.01, ***P<0.001, ****P<0.0001

To evaluate the effect of pH on the reductase activity, both RBC and hemolysate were incubated under hypoxic conditions and the release of NO was measured. Figure 5 shows that a decrease in pH to levels that have been reported in pathologic conditions increased the reductase capacity of both RBC and hemolysate. Lowering the pH resulted in a significant increase in NO release. The rate in the linear phase of NO release from RBC samples was 0.179 (R^2^ = 0.997) at pH 7.4, 0.285 (R^2^ 0.997) at pH 6.8, and 0.366 (R^2^ 0.998) at pH 5.5 (Figure 5A and 5B). For hemolysates these rates were 1.141 (R^2^ = 1.000) at pH 7.4, 1.411 (R^2^ = 1.000) at pH 6.8 and 1.467 (R^2^ = 1.000) at pH 5.5 (Figure 5C and 5D). These differences were not due to a different uptake of nitrite. RBC in an environment with a lower pH did not show differences in nitrite influx compared to RBC in a physiological environment (Figure 5E). Finally, To test whether other reported enzymes rather than hemoglobin in RBC and hemolysate samples, play a role in reducing nitrite to NO, as has been demonstrated at lower pH [10], we added the eNOS inhibitors N^ω^-Nitro-L-arginine methyl ester (L-NAME; 3 mM) and N^ω^-methyl-L-arginine acetate salt (L-NMMA; 3 mM) and the XOR inhibitor allopurinol (0.5 mM) to RBC and hemolysates at pH 5.5, prior to incubation with nitrite. None of the inhibitors showed a negative effect on the nitrite mediated NO release at any of the used concentrations, indicating that the NO release detected in our experiments did not originate from nitrite reduction by these enzymes in the RBC (Figure S8 in File S1)

### Nitrite reductase by RBC is temperature-dependent

**Figure 6:**
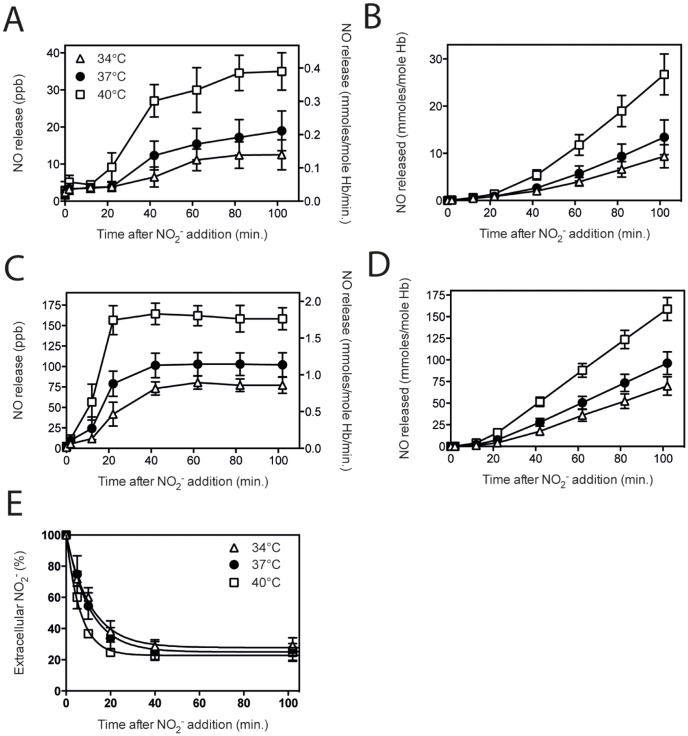
Hemoglobin-dependent NO release is temperature-mediated. Temperature increase to 40°C represents feverish conditions, and temperature decline to 34°C represent hypothermic conditions. When 5 mM nitrite was incubated at a lower temperature, RBC and hemolysate-mediated NO release was decreased compared to incubation at 37°C. Exposure to an elevated temperature resulted in a significant increase in NO release when compared to 37°C. NO release rate is depicted in A & C, and total release in time in B & D. Nitrite influx into RBC was faster with increasing temperatures, however, the total percentage of nitrite transported into the RBC from 40 minutes on, was equal at all three temperatures (E). A & B: RBC 34°C n = 3, Hb 11.7±0.3, 37°C n = 32 Hb 11.6±0.4 g/dL, 40°C n = 4, Hb 11.7±0.3 g/dL. C & D: Hemolysate; 34°C n = 4, Hb 11.6±0.3 g/dL, 37°C n = 29, Hb 11.6±0.3 g/dL, 40°C n = 5, Hb 11.7±0.3 g/dL, E: influx curve fits: 34°C y = 27.8+72.0*exp(−0.090*t); R^2^ = 0.996, 37°C y = 25.0+76.2*exp(−0.095*t); R^2^ = 0.996, 40°C y = 22.8+77.8*exp(−0.159*t); R^2^ = 0.997 Statistics: *RBC NO release rate*: 40°C vs. 37°C; * after 22 minutes, **** after 42–102 minutes, 40°C vs. 34°C; **** after 42–102 minutes, 37°C vs. 34°C; * after 42 minutes, and ** after 102 minutes. *RBC total NO release*: 40°C vs. 37°C; ** after 42 minutes and **** after 62–102 minutes, 40°C vs. 34°C; * after 42 and **** after 62–102 minutes, 37°C vs. 34°C; * after 82 minutes, and *** after 102 minutes. *Hemolysate NO release rate*: 40°C vs. 37°C; **** after 12–102 minutes, 40°C vs. 34°C; **** after 12–102 minutes, 37°C vs. 34°C; **** after 22 and 42 minutes, ** after 62 minutes, *** after 82 and 102 minutes. *Hemolysate total NO release*: 40°C vs. 37°C; * after 22 minutes, **** after 42–102 minutes, 40°C vs. 34°C; * after 22 minutes, **** after 42–102 minutes, 37°C vs. 34°C; ** after 42 minutes, *** after 62 minutes, and **** after 82 and 102 minutes. *Influx*: 34°C vs. 40°C; **** after 10 minutes, and * after 20 minutes, 37°C vs. 40°C; ** after 5 minutes, and *** after 10 minutes. *P<0.05, **P<0.01, ***P<0.001, ****P<0.0001

When the tonometer temperature was reduced to 34°C, to mimic hypothermic conditions, addition of 5 mM nitrite resulted in a significantly lower NO release (Figure 6A and 6B). When the tonometer temperature was increased to 40°C, to mimic feverish conditions, a significantly higher NO release was measured (Figure 6C and 6D). The rate in the linear phase of NO release from RBC samples was 0.129 (R^2^ = 0.993) at 34°C, 0.179 (R^2^ = 0.998) at 37°C, and 0.356 (R^2^ = 0.998) at 40°C. For hemolysates these rates were 0.868 (R^2^ = 1.000) at 34°C, 1.141 (R^2^ = 1.000) at 37°C, and 1.787 (R^2^ = 1.000) at 40°C. Nitrite influx into RBC was affected by temperature. At higher temperatures the influx is faster in the first 20 minutes. However, after 40 minutes these differences have disappeared and the total amount of nitrite taken up by RBC is the same (75% of nitrite available) for all three temperatures (Figure 6E). These differences in the initial uptake and the relative small fraction of nitrite converted to NO are highly unlikely to affect the NO release measured at different temperatures. Together these results show that both pH and temperature can affect the ability of RBC or hemolysate to generate NO and release it into the gas-phase.

### Increased nitrite reductase capacity of sickle red blood cells

**Figure 7:**
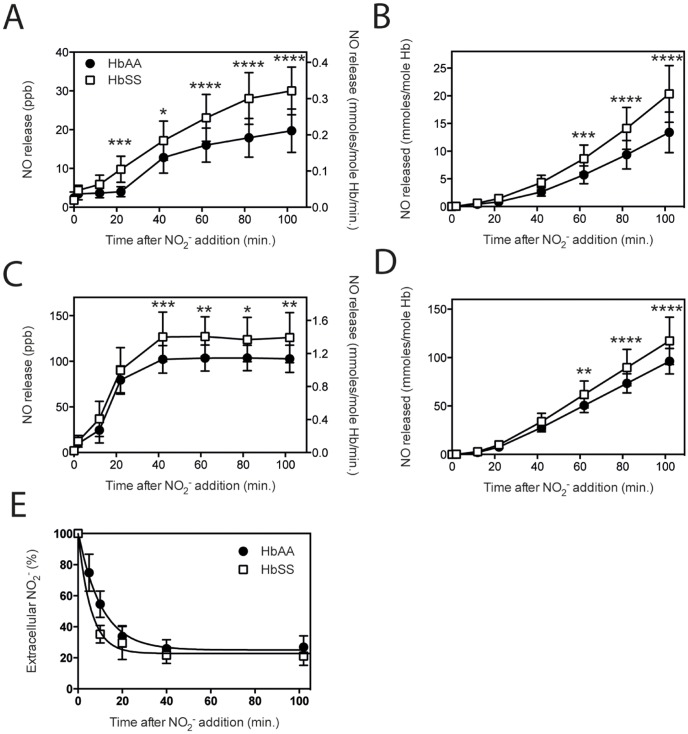
Improved nitrite reductase capacity of sickle red blood cells. The ability to reduce nitrite to NO by RBC and hemolysates from patients with homozygous sickle cell disease (HbSS) was compared to normal controls (HbAA). RBC from patients with SCD, showed superior NO release upon incubation with 5 mM nitrite under hypoxia, compared to normal controls (A & B). Also, when RBC were lysed and these hemolysates were incubated with nitrite under hypoxic conditions, HbSS containing specimen showed an increased NO release compared to hemolysates of normal controls (C & D). Nitrite influx into RBC was faster with increasing temperatures, however, the total percentage of nitrite transported into the RBC from 40 minutes on, was equal for RBC derived from normal and patients with sickle cell disease (E). A & B: RBC n = 10, Hb 12.1±1.1 g/dL, C & D: hemolysates n = 7, Hb 11.8±1.8 g/dL, E: influx curve fits: HbAA y = 25.0+76.2*exp(−0.095*t); R^2^ = 0.996, HbSS y = 22.8+77.1*exp(−0.171*t); R^2^ = 0.995, Statistics: *Influx*: HbSS vs. HbAA; **** after 10 minutes (5 min not done) *P<0.05, **P<0.01, ***P<0.001, ****P<0.0001.

Blood samples of homozygous SCD patients (HbSS) were used to evaluate the generation of NO and release into the gas-phase from nitrite. Incubation of sickle RBC, with the same hemoglobin concentration present in the incubation as normal HbAA controls, showed increased reductase capacity (Figure 7A-D). The rate in the linear phase of NO release from RBC or hemolysate samples from sickle cell patients was 0.268 (R^2^ = 0.994) and 1.390 (R^2^ = 1.000), as compared to normal controls, which showed 0.179 (R^2^ = 0.997) and 1.141 (R^2^ = 1.000), respectively. Influx of nitrite into sickle RBC seems slightly faster as compared to normal controls (Figure 7E). Nevertheless, it is unlikely that this slightly increased influx in the initial phase can account for the increased NO release. This is further confirmed by the increased release of NO by hemolysates, which are not affected by nitrite influx.

### Comparable nitrite reductase capacity of cord blood

**Figure 8:**
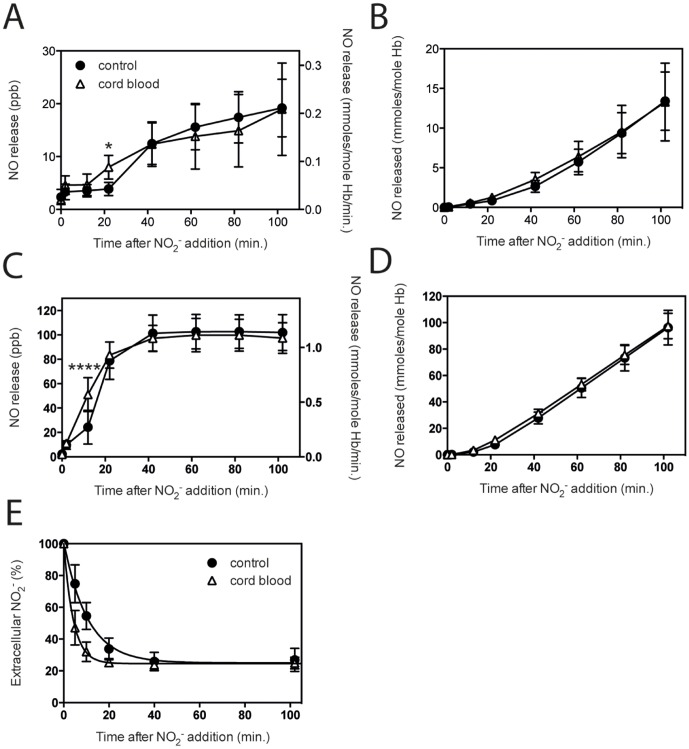
Comparable nitrite reductase capacity of cord blood. The ability to reduce nitrite to NO by RBC and hemolysates from cord blood, which typically contains a high concentration of fetal hemoglobin (HbF), was compared to normal controls (HbAA). Incubation with 5 mM nitrite, under hypoxic conditions at 37°C, showed a slightly increased NO release for umbilical cord derived blood samples when compared to normal controls for both RBC (A & B) and hemolysates (C & D). Transport of extracellular nitrite into the red cells was considerably faster in the first 40 minutes, however, the total percentage nitrite in the cellular fraction from 40 minutes on was equivalent, about 76% of totally added nitrite (E). A & B: RBC n = 7, Hb 11.8±0.1 g/dL, C & D: hemolysates: n = 7, Hb 11.8±0.1 g/dL, E: influx curve fits: control blood y = 25.0+76.2*exp(−0.095*t); R^2^ = 0.996, cord blood y = 24.5+75.5*exp(−0.238*t); R^2^ = 1.000 Statistics: *Influx*: cord blood vs. control blood; **** after 5 and 10 minutes, and * after 20 minutes. *P<0.05, ****P<0.0001

The umbilical cord samples used contained a significant percentage (84.8±4.3%) of fetal hemoglobin (HbF), which is comparable to previously published percentages [21]. RBC from cord blood also have a larger mean corpuscular volume (MCV), which was on average 90 fL for normal control and 102 fL for cord blood samples used. Samples were diluted to match the normal control RBC hemoglobin concentration (11–12 g/dL), in order to compare the effect of hemoglobin on the release of NO into the gas-phase. Both RBC and hemolysates from cord blood showed similar NO release when compared to adult blood samples with matching hemoglobin concentration (Figure 8). The rate in the linear phase of NO release from RBC (Figure 8A and 8B) or hemolysate (Figure 8C and 8D) samples from cord blood was 0.162 (R^2^ = 0.997) and 1.105 (R^2^ = 1.000), as compared to normal controls, which showed 0.179 (R^2^ = 0.997) and 1.141 (R^2^ = 1.000), respectively. The influx of nitrite was significantly faster into RBC from cord blood samples in the initial phase, but after 40 minutes the total percentage of added nitrite that was transported from the extracellular environment into the RBC was the same for controls and cord blood (Figure 8E).

## Discussion

The ability of hemoglobin to reduce nitrite to NO has been claimed to have a critical function in vascular physiology [20], [22], [23]. However, evaluating the reductase activity of RBC under conditions that mimic blood physiology is complex. Nitrite is rapidly oxidized to nitrate, affecting the levels of substrate available in the circulation. Nitrite has to be taken up by the RBC, before it can act as a substrate for the reductase reaction, and thus the process in which the nitrite crosses the RBC membrane will affect the reduction of nitrite to NO. Adding to the complexity is the chemistry of nitrite reactions with hemoglobin in its different states modulated by oxygen. The catalytic conversion to NO renders a product that will affect its own formation by its reaction with hemoglobin. Finally to be effective, NO needs to be able to exit the RBC and transfer to its site of action. This process implies a path where the product (NO) can and will react with both cytosolic and membrane components. Measurements of exhaled NO have been used to evaluate NO metabolism in patients [24]–[29], and we argued that a similar system in which NO is measured in the gas-phase formed from a RBC solution that mimics physiology would be useful to evaluate different conditions that may affect the reduction of nitrite to NO. In this study, we describe a highly reproducible method using simple technology to detect released NO from blood samples in a 2% BSA containing buffer, under various conditions at physiologically relevant hemoglobin concentrations. Evaluating the intact RBC at physiologic concentrations as the “enzymatic activity” to generate NO from nitrite, takes into account all steps in the process: uptake of substrate, conversion to NO inside the RBC, release of NO from the cells and surrounding buffer, and the changes in the RBC that result from this process. The closed environment and relatively small headspace of the tonometer, allowed exposure of blood samples to gas with a defined composition and continuous flow at a constant rate (to prevent accumulation in the tonometer). Nitric oxide released from the sample in the tonometer, present in the outflow gas, was collected in mylar balloons and subsequently detected by chemoluminescence in a Nitric Oxide Analyzer. This system is based on the method that is used in clinical studies to measure NO in exhaled air of patients (exNO or eNO) [24], [29], [30]. To study the enzymatic reductase capacity of RBC we used both intact RBC and hemolysates at a physiological hemoglobin concentration. However, in order to obtain detectable NO levels other factors, such as nitrite concentration, were not as seen in normal physiology. The main objective was to acquire information on the kinetics of RBC to release NO from nitrite under different conditions. These kinetics include both uptake of nitrite into the cell, generation of NO and transport of NO to the gas-phase (generation, capture and diffusion). Comparison of RBC with hemolysate allows assessment of the presence or absence of an intact membrane on these pathways.

Several studies have investigated the uptake of nitrite by RBC under hypoxia. Transport of nitrite across the RBC membranes is a crucial step in intracellular nitrite-to-NO conversion. It has been shown previously that nitrite rapidly permeates and equilibrates across the RBC membrane and then continues to enter the RBC as a result of intracellular nitrite consumption upon reacting with hemoglobin, and is dependent on fractional saturation [31], [32]. The mechanism by which extracellular nitrite crosses the membrane, has been proposed to be both via HNO_2_ diffusion through the lipid bilayer and via the anion exchanger 1 (AE1) [32], [33]. Our results show that nitrate does not enter the RBC and confirm that the RBC act as a sink for nitrite in a diffusion two pool model when nitrite is added to RBC in 2% BSA containing buffer. The total intracellular RBC volume present in most experiments sample was approximately 175 µl (35% hematocrit) in a total volume of 500 µl. Free diffusion kinetics predict 65% extracellular nitrite and 35% intracellular nitrite, at equilibrium. We found that after 100 minutes only 26.7±7.4%, rather than 65%, remained in the extracellular compartment, under strict hypoxic conditions, thus excluding conversion of nitrite to nitrate.

While the central reaction is the generation of NO, our approach does not give direct information on the reaction of the conversion of nitrite to NO, as the complete pathway including release into the gas-phase is measured. Hence we describe the results as release of NO rather than generation of NO. We found that our setup was able to sensitively measure the release of NO from a RBC suspension at physiologic relevant hematocrit. To treat the intact RBC as the “enzymatic active entity” and gain information in the linear phase of NO release, the nitrite concentration needed to be much higher than plasma nitrite concentration reported (150 to 1,000 nM [22]). We aimed to study the reductase capacity of RBC as a whole. Dramatically reducing the hematocrit below physiologic concentrations allows a lower nitrite concentration, reflecting conditions as were reported before [10], [12], [20], [22], [34]. Importantly, the ratio of nitrite relative to RBC or hemoglobin in virtually all reported studies is higher than can be expected *in vivo*. However, either hematocrit has to be below physiologic conditions, or nitrite has to be high to gain relevant information on the ability of the RBC to convert nitrite to NO. To compare the ability of RBC to release NO under different conditions, a linear phase in the formation of substrate to product needs to be achieved, as is the case with assessments of metabolic enzyme reactions. This led us to choose for a physiologic relevant hematocrit with relatively high nitrite. For our experiments we used a hemoglobin concentration in the physiological range of 12 g/dL, which is 1.86 mM hemoglobin and 7.45 mM heme (each Hb tetramer contains 4 hemes entities). For each run we used 500 µl sample volume, which is 0.93 µmole hemoglobin and 3.73 µmole heme. Under strict anaerobic conditions, one nitrite molecule converts two hemoglobin molecules to one metHemoglobin and one iron-nitrosyl hemoglobin [12]. A nitrite concentration of 5 mM leads to a molar heme to nitrite ratio of approximately 7.5∶5.0, thus nitrite is not expected to be fully consumed. Our approach allowed us to measure generation of NO in the linear phase and the accumulation of the total amount of NO release into the gas-phase over time, without markedly changing the oxygen affinity (Figure S10 in File S1). This provided data on the percentage of nitrite that was released in the form of NO into the gas-phase. After 100 minutes, 0.33% of nitrite added was released as NO using RBC. For hemolysate samples this was 3.33%. The total amount of nitrite reduced to NO cannot be determined from the released NO, as part of the product (NO) reacts with components in the mixture and will not be released in the gas-phase. This seems apparent in approximately the first 20–30 minutes of the reaction where the release of both RBC and hemolysate is progressing at an apparently slow rate. In the case of intact RBC this is further complicated with the rate of nitrite uptake and possibly NO transport out of the cell. However after the first 20 minutes the rate at which NO is released reaches a constant rate. At that time an equilibrium is reached for nitrite uptake and it can be speculated that most of the sites have the potential to react with nitrite or its metabolites are saturated. Interestingly, simple stoichiometry and the rate of NO release cannot explain our findings. Apparently, NO slowly “leaks” from the cell after it is formed from nitrite but our study does not allow us to conclude to which entity NO is bound or released from.

In all our experiments, RBC were compared to hemolysates in order to study the effects of hemoglobin compartmentalization and density, and the role of the intact red cell membrane in release of NO and influx of nitrite. Interestingly, hemolysates showed remarkably higher NO release in all experiments. Using normal blood at 37°C and neutral pH, the rate of release from hemolysate was about 6.5 times higher (linear rate: 1.141) as compared to RBC (linear rate: 0.179). This difference cannot be attributed to the 25% higher availability of nitrite.

In contrast to most reported studies that use either isolated purified hemoglobin or very low RBC concentrations our data render results that include the potential effects of important parameters such as hemoglobin density, the role of membrane and presence of other enzymes that exhibit nitrite-to-NO reduction activity. The role of RBC in nitrite regulated NO-dependent hypoxic vasodilation was also previously studied by Crawford and colleagues [20]. They also show that hemoglobin functions as an enzyme capable of nitrite reduction under hypoxic conditions. In their study, NO generation is detected through measuring vasodilation in rabbit aortas and RBC were exposed to much lower nitrite concentrations but also at a much lower hematocrit (0.3%). Detection of NO release from hemolysate samples diluted to 1% (0.2 g/dL hemoglobin), instead of approximately 35% (12 g/dL hemoglobin), allowed us to also decrease nitrite concentration at least 10x (Figure S9 in File S1) and still obtain detectable NO release levels. In addition, RBC-mediated hypoxic nitrite reduction was also studied in a model in which isolated rat lungs were perfused [35]. In that study, it is shown that hypoxic pulmonary vasoconstriction (HPV) was inhibited at low micromolar nitrite concentrations when RBC concentration was subphysiological (1% hematocrit) but not at higher RBC concentrations (15% hematocrit).

The tetrameric conformation of hemoglobin is key in the regulation of carbon dioxide and oxygen binding or dissociation. Allosteric transition between the R(elax)-state (high oxygen affinity) and T(ense)-state (low oxygen affinity), however, has not only been acknowledged to play a primary role in carbon dioxide-oxygen regulation but has been shown to be important in vascular NO regulation and signaling [36]–[38]. R-state hemoglobin has a low heme redox potential which favors distribution of electrons to nitrite leading to an increased reactivity with the unliganded ferrous hemes [13]. Several ligands have been described to promote T-state (deoxyhemoglobin) to R-state transition including oxygen, carbon monoxide, NO and oxidation to methemoglobin. In our system, incubation of oxyhemoglobin with nitrite showed no NO release at all. When hypoxic conditions were changed to normoxic conditions 1 hour after addition of nitrite, dramatic loss of NO released from tonometer after 10 minutes exposure to air, which is recovered for about 75% when hypoxia is restored for 10 minutes. The lack of a complete recovery may have different reasons. The cycles between air and nitrogen will generate methemoglobin, and we show that pure hemoglobin (mainly methemoglobin) has a lower reductase activity. A fraction of nitrite will be oxidized to nitrate lowering the available substrate and possibly nitrate may affect the reduction rate. Addition of nitrate to hemolysate has no effect and although addition of nitrite increases the rate of NO release, it is partially recovered to its original level. This suggests that the change in substrate is not responsible for the lack of recovery but rather a change in reductase activity.

Stamler and co-workers have reported that under low pO_2_, NO is bound to heme in hemoglobin in a T-state, and upon oxygenation, NO is transferred from heme-bound NO to thiol groups of hemoglobin (Cys β93; SNO-Hb), which is transformed to an oxy or R-state, and finally when in circulation the pO_2_ drops again, the promotion of hemoglobin to a T-state conformation, releases NO [39], [40]. Our cycles between air and nitrogen do not show bursts of NO released into the gas-phase. The chemical biology of nitrite reactions and the R- and T-state of hemoglobin was reviewed in detail by Gladwin et al. [13]. Since we performed our reactions with intact RBC rather than pure hemoglobin, we did not aim to specifically test R-state hemoglobin catalysis in this complex system. Modification of hemoglobin by CO, only slightly altered the release pattern of NO from the cells. On the other hand, the addition of NO using a NO donor appears to affect the release of NO from the cells at the start of the reaction as can be predicted by Gladwin et al. [13]. However, one could also argue that the release is facilitated by the reaction of this added NO with those components that capture NO at the beginning of the reaction, leading to an increase in NO release as these sites are saturated.

To investigate clinical relevance of our method we added nitrite to RBC at different (patho)physiological conditions. Clinically, a decreased peripheral blood pH is found in several conditions such as sepsis [41] and tissue ischemia [42]. Webb and colleagues investigated the role of potential nitrite reductases, other than hemoglobin, present in RBC [10]. They show that xanthine oxidoreductase (XOR) and endothelial nitric oxide reductase (eNOS), both present in RBC membranes, contribute to RBC-mediated NO generation. RBC XOR-mediated nitrite reductase activity was mainly seen under acidic conditions. Interestingly, under hypoxic, physiologic and acidic conditions, addition of eNOS-inhibitors L-NAME and L-NMMA to RBC showed a significant reduction of NO generation upon incubation with nitrite. In our studies we did not see reduced NO release from blood samples caused by XOR inhibitor allopurinol or eNOS inhibitors L-NAME and L-NMMA at decreased pH. Temperature affected hemoglobin reductase capacity significantly. Nitrite reduction under hypothermic temperature (34°C) showed a decrease in NO release, whereas incubation at hyperthermic, feverish temperature (40°C) increased nitrite-to-NO reduction when compared to 37°C. This is in agreement with the temperature regulation in vertebrates; blood vessels are smaller and blood flow is reduced under cooler circumstances (to conserve heat) and blood vessels are more dilated under warmer temperatures (to give off heat). However, the role of hemoglobin mediated NO generation has not been investigated to our knowledge.

Differences in nitrite reductase activity have been reported for different types of hemoglobin. Roche and co-workers showed that hemoglobin E has a reduced nitrite reductase capacity when compared to hemoglobin A [43]. Based on the change in hemoglobin species rather than NO itself. NO metabolism is very important in sickle cell disease. We, and others have shown that it is dysregulated [17], [44], and the measurement of exhaled NO has been used in clinical studies in sickle cell disease [24]. Both decreased hematocrit and increased free plasma hemoglobin have been described to contribute to the vaso-occlusive events found in SCD, leading to painful episodes and possible organ damage [44], [45]. Blood from homozygous sickle cell patients slightly increased nitrite reductase capacity, when sickle and control blood was compared at the same hemoglobin concentration either as RBC or hemolysate. This confirms the findings by Grubina et al. [46]. Sickle cell patient typically show lower hemoglobin concentration than normal controls. Based on our experiments at different hematocrits, this would enhance the release of NO. Plasma free hemoglobin is increased in patients with SCD as a consequence of hemolysis. On the one hand this will lead to NO scavenging [44], but since hemolysate is effective in reducing nitrite, this may paradoxically lead to increased NO formation. Fetal cells collected from the umbilical cord RBC are unique with respect to their volume, membrane composition, biophysical properties, metabolism, enzyme profile, and hemoglobin [47]. One of the most important physiological differences is the high fetal hemoglobin (HbF) content, and our data show that fetal cells exhibit an initial slight increase in NO release from nitrite reduction. Hence, hemoglobin with a left or right shifted oxygen affinity curve as compared to normal hemoglobin can exhibit changes in NO release from nitrite reduction. An increase in fetal hemoglobin in hemoglobinopathy patients treated with compounds like hydroxyurea may therefore increase the formation of NO from nitrite. Futhermore, formed NO *in vivo* likely also interacts with other high affinity receptors such as soluble guanylyl cyclase (sGC) and cytochrome C oxidase. Recent studies have shown that this has important biological effects such as platelet inactivation and consequent prolonged bleeding times [48], [49].

In summary, we present a novel approach to evaluate the ability of RBC to generate NO from nitrite, released into the gas-phase. The approach described in this paper is valid to better understand the complete complex process by which the RBC is able to lead to the formation of NO from nitrite. While it provides information on the ability to generate NO, caution is required to apply these findings directly to physiology. Methods that use purified hemoglobin [14], [34], or physiologically low hematocrits [10], [20] can be criticized to be non physiological. In contrast, our approach uses intact or hemolyzed RBC at physiologic relevant concentrations and provides the opportunity to evaluate a complete RBC system as physiologic hematocrit. However in order to compare different conditions and reach a linear phase of NO release, higher, non-physiological nitrite concentrations are required. Deem et al. show the nitrite reductase capacity of hemoglobin under hypoxic conditions but suggest that insufficient NO escapes from RBC at physiological submicromolar nitrite concentrations [35]. Indeed with a physiologic hematocrit and a low nitrite concentration we do not see the release of NO in the gas-phase. However, the goal of this study was to evaluate the RBC as an enzymatic entity to generate NO from nitrite, and the substrate available should not be limiting. This approach, allows experimental evaluation of blood modifications that improve reductase activity to contribute in modulating vascular tone in a variety of pathological conditions including hemoglobinopathies, and conditions associated with hemolysis and ischemia-reperfusion injury [22], [50]–[53]._ENREF_24 Improving the NO reductase capacity of RBC combined with increased nitrite levels by infusion could be a potential novel therapeutic strategy.

## Supporting Information

File S1 **Figure S1. NO donors validate applicability of NO detection method.** Two NO donors, diethylamine (DEA) NONOate, a rapid dissociating molecule and Spermine (Sper) NONOate, a much slower dissociating NO donor (release half-life at physiological conditions 2.1 and 39 min, respectively) were tested in the experimental set up. The NO donors were diluted in HBS containing 2% BSA (HBA2%). The reasons for addition of 2% BSA is two fold; first to mimic the main protein component of plasma to create ‘artificial plasma’ and secondly because it acts protectively to prevent hemolysis (due to the shear force induced by the stirring of the cuvette in the tonometer) in case intact RBC are present. Free NO reacts with the free cysteines of BSA to form S-nitroso BSA (S-NO-BSA). Comparison of HBA 2% with HBS indeed showed some decrease in NO released into the headspace of the tonometer (data not shown) until total nitrosylation was reached. When a final concentration of either 20 or 50 µM DEA NONOate under constant nitrogen flow was measured, an initial high spike of about 300 ppb and 750 ppb NO, respectively, collected the first 2 minutes after addition of DEA NONOate, could be detected (A). After 5 minutes the NO released from the tonometer was dramatically decreased for both concentrations of DEA NONOate. At 10 minutes and beyond, NO released from the tonometer was negligible. When Sper NONOate was added under nitrogen flow, either 50 or 250 µM, comparable to 20 and 50 µM DEA NONOate NO release could be detected (170 ppb and 750 ppb, respectively). Every 10 minutes NO release was detected up to 100 minutes, first order NO release could be detected (B). For both NONOates, when instead of nitrogen, air was used to flow through the tonometer chamber identical results were obtained (data not shown). Additionally, when the highest concentration of either NONOate was added to hemoglobin containing samples, hardly any (∼20 ppb) of the in HBA 2% measured released NO could be detected (C). **Figure S2. Nitrite influx at different nitrite and different RBC concentrations.** (A) When 1 mM nitrite is added to RBC, at 11–12 g/dL concentration, virtually all nitrite is transported into the red cell fraction. Interestingly, addition of 10 mM nitrite to RBC led to only about 50% of total nitrite transport into the red cells fraction, indicating that RBC do have a limit for nitrite uptake. (B) Extracellular nitrite levels RBC for samples with a hemoglobin concentration of 5.0, 8.4, 11.9 and 16.8 g/dL (equivalent to 15, 25, 35 and 50% total intracellular RBC volume (hematocrit), respectively), showed that approximately 65, 40, 25 and 5% of the total nitrite added to these samples, remained in the extracellular compartment after 60 minutes. **Figure S3. Partial replacement of intact RBC by hemolysed RBC improves NO release.** When 5 and 10% of the RBC sample is replaced by hemolysed RBC sample (so total hemoglobin concentration remains equal), increased NO release is detected in a percentage-dependent fashion (A & B). Transport of nitrite into RBC was not affected by the presence of hemolysate (C). A–C: 0% hemolysis n = 32, 5% hemolysis n = 5, 10% hemolysis n = 5. Statistics: *NO release rate*: 0% vs. 5%; * after 22 and 102 minutes, *** after 42 minutes, and ** after 62 and 82 minutes, 0% vs. 10%; ** after 12 minutes, and **** after 22–102 minutes, 5% vs. 10%; * after 62–102 minutes. *total NO release*: 0% vs. 5%; *** after 62 minutes, and **** after 82 and 102 minutes, 0% vs. 10%; ** after 42 minutes, and **** after 62–102 minutes, 5% vs. 10%; ** after 82 minutes, and *** after 102 minutes. *P<0.05, **P<0.01, ***P<0.001, ****P<0.0001. **Figure S4. Nitrite to nitrate oxidation when performed under normal air.** Nitrite and nitrate were both measured during an entire run under air with an added final nitrite concentration of 5 mM. Nitrite is gradually converted to nitrate in the presence of oxygen. **Figure S5. Hemolysate exposure to 5 mM nitrite for 1 hour under hypoxic (N_2_) conditions followed by 2 minute air-N_2_ cycles.** Alternate 2 minutes cycles of air and N_2_ demonstrated that shorter exposure to nitrogen rather than air decreased the NO release signal. **Figure S6. Nitrite reductase capacity of purified commercial hemoglobin**: Purified commercially purchased hemoglobin was diluted with HBS-BSA 2% to 12 g/dL, to directly compare NO release to RBC and hemolysates at similar concentrations (A and B). When comparing commercial hemoglobin with concentrations of 4, 8, 12 and 16 g/dL, incubated with 5 mM nitrite under strict hypoxia, a concentration-dependent effect was seen alike we have seen for hemolysates (C, D and E). **Figure S7. Nitrite transport into RBC saturated with NO and CO, under hypoxia.** Both pre-saturation with NO (by addition of excess NO donor) or CO (by pre-exposure to CO) did not result in significantly changed nitrite influx kinetics when compared normal RBC under N_2_ (Figure 5A and 5B). **Figure S8. eNOS and XOR inhibitors do not affect NO release from RBC and hemolysates at pH 5.5.** Endothelial nitric oxide synthase inhibitors L-NAME (0.3 & 3 mM) and L-NMMA (0.3 & 3 mM) and xanthine oxidoreductase inhibitor allopurinol (0.1 & 0.5 mM) were added to RBC and hemolysate samples at pH 5.5, to study their possible contribution to nitrite reduction leading to NO release. For none of the inhibitors, either added to RBC or hemolysate samples, NO release was decreased, suggesting all NO release measured originated from hemoglobin reduction of nitrite (A–D). **Figure S9. NO release by 1% hematocrit hemolysates after addition of a range of nitrite concentrations.** NO release under hypoxia, when hemolysates were diluted to 1% hematocrit ( = 0.2 g/dL), reduced minimum nitrite concentration at which NO could be detected. A ten times lower than normal nitrite concentration (0.5 mM) still showed detectable NO levels released from the tonometer. **Figure S10. Oxygen affinity curves of RBC in 2% BSA-HBS during continuous incubation with N_2_.**
(DOCX)
