# Supplementary material for: The Capacity of Red Blood Cells to Reduce Nitrite Determines Nitric Oxide Generation under Hypoxic Conditions
Source: PLoS One. 2014 Jul 9;9(7):e101626. doi: 10.1371/journal.pone.0101626 (PMC4090171; doi:10.1371/journal.pone.0101626)
Supplement: File S1 — Figure S1. NO donors validate applicability of NO detection method. Two NO donors, diethylamine (DEA) NONOate, a rapid dissociating molecule and Spermine (Sper) NONOate, a much slower dissociating NO donor (release half-life at physiological conditions 2.1 and 39 min, respectively) were tested in the experimental set up. The NO donors were diluted in HBS containing 2% BSA (HBA2%). The reasons for addition of 2% BSA is two fold; first to mimic the main protein component of plasma to create ‘artificial plasma’ and secondly because it acts protectively to prevent hemolysis (due to the shear force induced by the stirring of the cuvette in the tonometer) in case intact RBC are present. Free NO reacts with the free cysteines of BSA to form S-nitroso BSA (S-NO-BSA). Comparison of HBA 2% with HBS indeed showed some decrease in NO released into the headspace of the tonometer (data not shown) until total nitrosylation was reached. When a final concentration of either 20 or 50 µM DEA NONOate under constant nitrogen flow was measured, an initial high spike of about 300 ppb and 750 ppb NO, respectively, collected the first 2 minutes after addition of DEA NONOate, could be detected (A). After 5 minutes the NO released from the tonometer was dramatically decreased for both concentrations of DEA NONOate. At 10 minutes and beyond, NO released from the tonometer was negligible. When Sper NONOate was added under nitrogen flow, either 50 or 250 µM, comparable to 20 and 50 µM DEA NONOate NO release could be detected (170 ppb and 750 ppb, respectively). Every 10 minutes NO release was detected up to 100 minutes, first order NO release could be detected (B). For both NONOates, when instead of nitrogen, air was used to flow through the tonometer chamber identical results were obtained (data not shown). Additionally, when the highest concentration of either NONOate was added to hemoglobin containing samples, hardly any (∼20 ppb) of the in HBA 2% measured released NO could be detected (C). F [file pone.0101626.s001.docx]

File S1: supporting information: Manuscript M. Fens et al.: “The capacity of red blood cells to reduce nitrite determines nitric oxide generation under hypoxic conditions”

**B**

**A**

**C**

**Figure S1. NO donors validate applicability of NO detection method.** Two NO donors, diethylamine (DEA) NONOate, a rapid dissociating molecule and Spermine (Sper) NONOate, a much slower dissociating NO donor (release half-life at physiological conditions 2.1 and 39 min, respectively) [[1](#_ENREF_1)] were tested in the experimental set up. The NO donors were diluted in HBS containing 2% BSA (HBA2%). The reasons for addition of 2% BSA is two fold; first to mimic the main protein component of plasma to create ‘artificial plasma’ and secondly because it acts protectively to prevent hemolysis (due to the shear force induced by the stirring of the cuvette in the tonometer) in case intact RBC are present. Free NO reacts with the free cysteines of BSA to form S-nitroso BSA (S-NO-BSA) [[2](#_ENREF_2),[3](#_ENREF_3)]. Comparison of HBA 2% with HBS indeed showed some decrease in NO released into the headspace of the tonometer (data not shown) until total nitrosylation was reached. When a final concentration of either 20 or 50 μM DEA NONOate under constant nitrogen flow was measured, an initial high spike of about 300 ppb and 750 ppb NO, respectively, collected the first 2 minutes after addition of DEA NONOate, could be detected (A). After 5 minutes the NO released from the tonometer was dramatically decreased for both concentrations of DEA NONOate. At 10 minutes and beyond, NO released from the tonometer was negligible. When Sper NONOate was added under nitrogen flow, either 50 or 250 μM, comparable to 20 and 50 μM DEA NONOate NO release could be detected (170 ppb and 750 ppb, respectively). Every 10 minutes NO release was detected up to 100 minutes, first order NO release could be detected (B). For both NONOates, when instead of nitrogen, air was used to flow through the tonometer chamber identical results were obtained (data not shown). Additionally, when the highest concentration of either NONOate was added to hemoglobin containing samples, hardly any (~20 ppb) of the in HBA 2% measured released NO could be detected (C).

**B**

**A**

**Figure S2. Nitrite influx at different nitrite and different RBC concentrations.** (A) When 1 mM nitrite is added to RBC, at 11-12 g/dL concentration, virtually all nitrite is transported into the red cell fraction. Interestingly, addition of 10 mM nitrite to RBC led to only about 50% of total nitrite transport into the red cells fraction, indicating that RBC do have a limit for nitrite uptake. (B) Extracellular nitrite levels RBC for samples with a hemoglobin concentration of 5.0, 8.4, 11.9 and 16.8 g/dL (equivalent to 15, 25, 35 and 50% total intracellular RBC volume (hematocrit), respectively), showed that approximately 65, 40, 25 and 5% of the total nitrite added to these samples, remained in the extracellular compartment after 60 minutes.

**
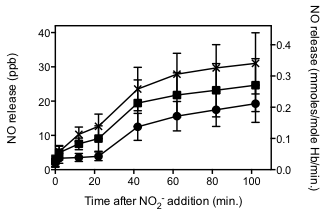
**

**B**

**C**

**A**


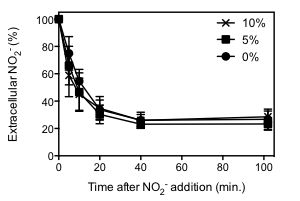

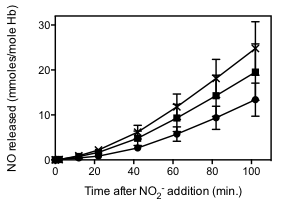


**
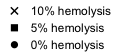
**

**Figure S3. Partial replacement of intact RBC by hemolysed RBC improves NO release.** When 5 and 10% of the RBC sample is replaced by hemolysed RBC sample (so total hemoglobin concentration remains equal), increased NO release is detected in a percentage-dependent fashion (A & B). Transport of nitrite into RBC was not affected by the presence of hemolysate (C).

A-C: 0% hemolysis n=32, 5% hemolysis n=5, 10% hemolysis n=5.

Statistics: *NO release rate*: 0% vs. 5%; * after 22 and 102 minutes, *** after 42 minutes, and ** after 62 and 82 minutes, 0% vs. 10%; ** after 12 minutes, and **** after 22-102 minutes, 5% vs. 10%; * after 62-102 minutes. *total NO release*: 0% vs. 5%; *** after 62 minutes, and **** after 82 and 102 minutes, 0% vs. 10%; ** after 42 minutes, and **** after 62-102 minutes, 5% vs. 10%; ** after 82 minutes, and *** after 102 minutes.

**P*<0.05, ***P*<0.01, ****P*<0.001, *****P*<0.0001

**Figure S4. Nitrite to nitrate oxidation when performed under normal air.** Nitrite and nitrate were both measured during an entire run under air with an added final nitrite concentration of 5 mM. Nitrite is gradually converted to nitrate in the presence of oxygen.

**B**

**A**

**Figure S5. Hemolysate exposure to 5 mM nitrite for 1 hour under hypoxic (N_2_) conditions followed by 2 minute air-N_2_ cycles.** Alternate 2 minutes cycles of air and N_2_ demonstrated that shorter exposure to nitrogen rather than air decreased the NO release signal.

****

**
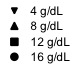
****
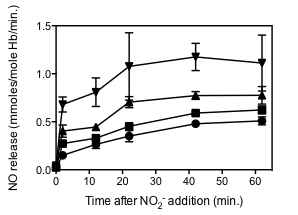

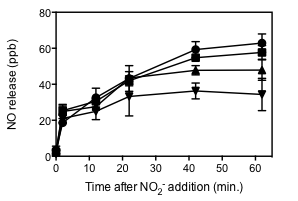

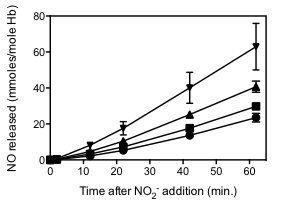
**

**C**

**B**

**A**

**E**

**D**

**Figure S6. Nitrite reductase capacity of purified commercial hemoglobin:** Purified commercially purchased hemoglobin was diluted with HBS-BSA 2% to 12 g/dL, to directly compare NO release to RBC and hemolysates at similar concentrations (A and B). When comparing commercial hemoglobin with concentrations of 4, 8, 12 and 16 g/dL, incubated with 5 mM nitrite under strict hypoxia, a concentration-dependent effect was seen alike we have seen for hemolysates (C, D and E).

**A**

**B**

****

**Figure S7. Nitrite transport into RBC saturated with NO and CO, under hypoxia.** Both pre-saturation with NO (by addition of excess NO donor) or CO (by pre-exposure to CO) did not result in significantly changed nitrite influx kinetics when compared normal RBC under N_2_ (Figure 5A and 5B).

**B**

**A**

**
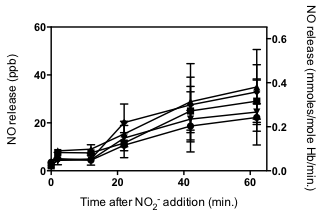

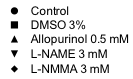

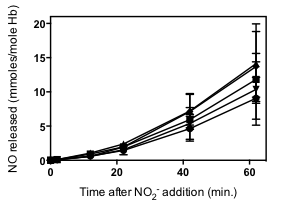

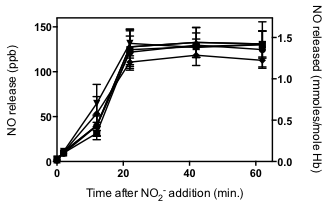

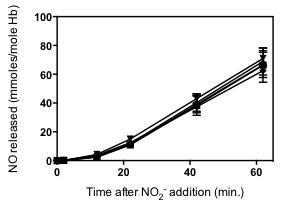
**

**C**

**D**

**Figure S8. eNOS and XOR inhibitors do not affect NO release from RBC and hemolysates at pH 5.5.** Endothelial nitric oxide synthase inhibitors L-NAME (0.3 & 3 mM) and L-NMMA (0.3 & 3 mM) and xanthine oxidoreductase inhibitor allopurinol (0.1 & 0.5 mM) were added to RBC and hemolysate samples at pH 5.5, to study their possible contribution to nitrite reduction leading to NO release. For none of the inhibitors, either added to RBC or hemolysate samples, NO release was decreased, suggesting all NO release measured originated from hemoglobin reduction of nitrite (A-D).

**
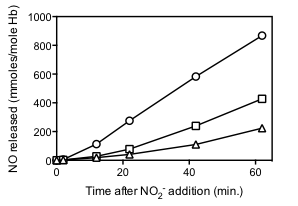

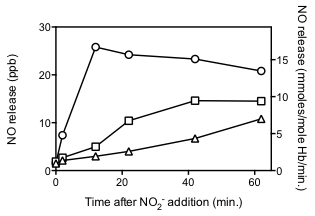
**

**B**

**A**


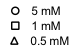


**Figure S9.** **NO release by 1% hematocrit hemolysates after addition of a range of nitrite concentrations.** NO release under hypoxia, when hemolysates were diluted to 1% hematocrit (= 0.2 g/dL), reduced minimum nitrite concentration at which NO could be detected. A ten times lower than normal nitrite concentration (0.5 mM) still showed detectable NO levels released from the tonometer.


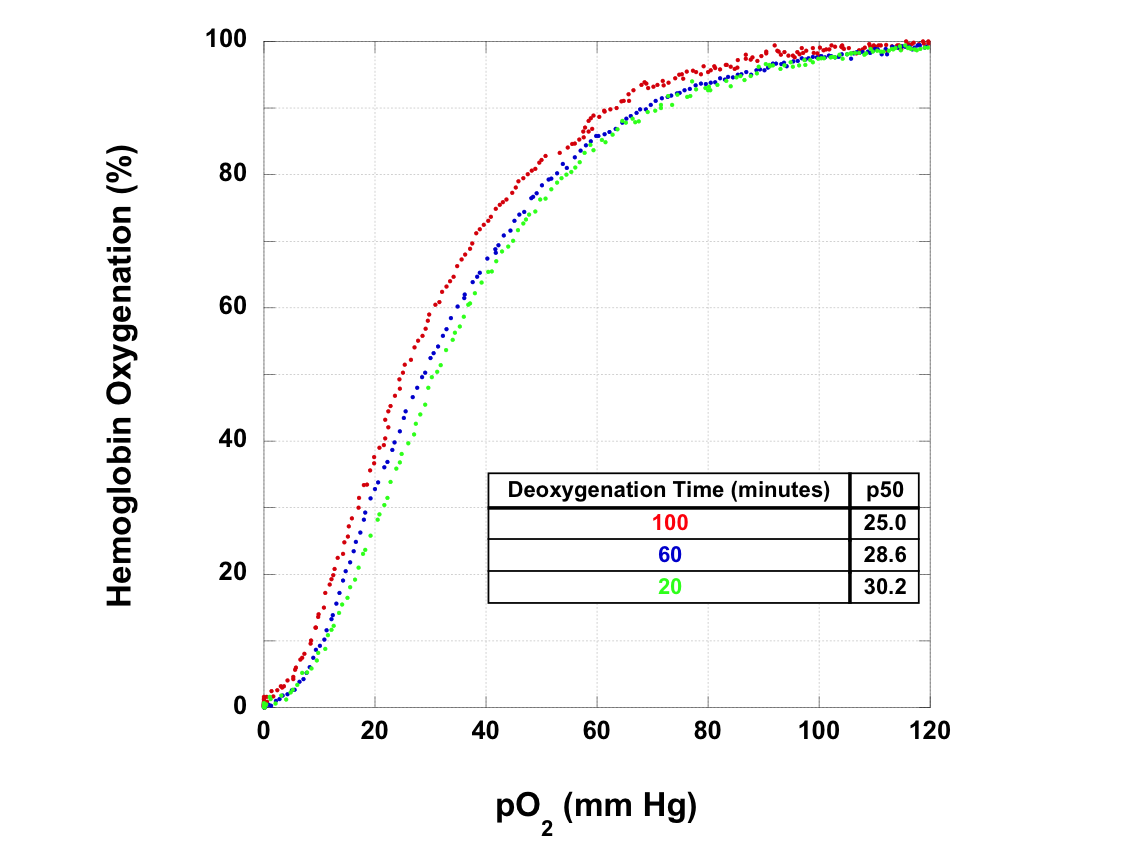


**Figure S10. Oxygen affinity curves of RBC in 2% BSA-HBS during continuous incubation with N_2_.**

**References**

1. Thomas DD, Miranda KM, Espey MG, Citrin D, Jourd'heuil D, et al. (2002) Guide for the use of nitric oxide (NO) donors as probes of the chemistry of NO and related redox species in biological systems. Methods Enzymol 359: 84-105.

2. Stamler JS, Simon DI, Osborne JA, Mullins ME, Jaraki O, et al. (1992) S-nitrosylation of proteins with nitric oxide: synthesis and characterization of biologically active compounds. Proc Natl Acad Sci U S A 89: 444-448.

3. Stamler JS, Jaraki O, Osborne J, Simon DI, Keaney J, et al. (1992) Nitric oxide circulates in mammalian plasma primarily as an S-nitroso adduct of serum albumin. Proc Natl Acad Sci U S A 89: 7674-7677.
